# Gastric ectopic pyloric opening: an unusual case

**DOI:** 10.1007/s00276-019-02276-x

**Published:** 2019-07-01

**Authors:** Bei Lu, Lili Yang

**Affiliations:** Department of Radiology, People’s Hospital of Xingtai City, No. 16 Hongxing Street, Xingtai, 054031 Hebei China

**Keywords:** Abnormality of pylorus, Gastric ectopic, Pyloric ectopic

## Abstract

Stomach is the most dilated part of the digestive tube. The shape of the stomach could vary frequently without any clinical symptoms. Abnormality of pylorus including double pylorus and congenital pyloric stenosis has been reported but pyloric ectopic opening has not been reported before. We found a rare case of pyloric ectopic opening in the stomach body with a “hammer” shape stomach in a 72-year-old man. The patient complained of upper left abdominal with no past medical history or surgery history. The double-contrast examination showed a “hammer” shape stomach, with the pylorus opening high at the lesser curvature and enlarged distal end of the stomach. The gastrointestinal endoscopy showed that the pyloric antrum was approximately 3 cm below the cardia with a round and poor functioning opening. No obvious abnormalities in the bulb and descending part of the duodenum were observed. A large ulcer with whitish exudate covering the base was found on the posterior wall. Histological examination of the ulcer showed broken mucosal glands with atypical hyperplasia and focal carcinogenesis. This case shows a probably congenital pyloric ectopic opening in the gastric body with a “hammer”-shaped stomach, adding a new gastric morphological variation.

## Background

Stomach is the most dilated part of the digestive tube. The stomach occupies the epigastric, left hypochondriac and umbilical regions of the abdomen [6]. Three types of stomach may persist clinically, which are hypersthenic, sthenic and hyposthenic types [4]. Sthenic type with a “J” shape is the most common type in living individuals.

Shape and position of the stomach can vary greatly with or without any physiological disturbances. Burdan et al. classified the anatomical variations of the stomach in five primary groups based on the radiological and historical data, which are abnormal position along longitudinal or horizontal axis, abnormal shape, abnormal stomach connections, and mixed form [2]. Among the variations of stomach, abnormality of pylorus is not commonly reported. One of the most commonly reported abnormalities of pylorus is congenital pyloric stenosis [13]. Few relevant reports to our knowledge about the pyloric ectopic opening have been reported. Here, we report a rare case of pyloric ectopic opening in the stomach body.

## Case presentation

### Clinical summary

A 72-year-old man complained of upper left abdomen discomfort for 3 weeks and aggravated for 2 days. No complaint of nausea, vomiting, abdominal pain, weight change, abdominal pain, diarrhea or other digestive disorder symptoms was reported. He was admitted to the gastroenterology department on December 21, 2015. The patient denied any past medical history or past surgery history. He had never smoked, and drunk alcohol only occasionally.

On examination, the patient was in good health. No abnormality in vital sign was detected. No other congenital abnormality was noticed during physical examination. His abdomen was non-tender, and no palpable mass was found.

### Double-contrast examination

**Fig. 1:**
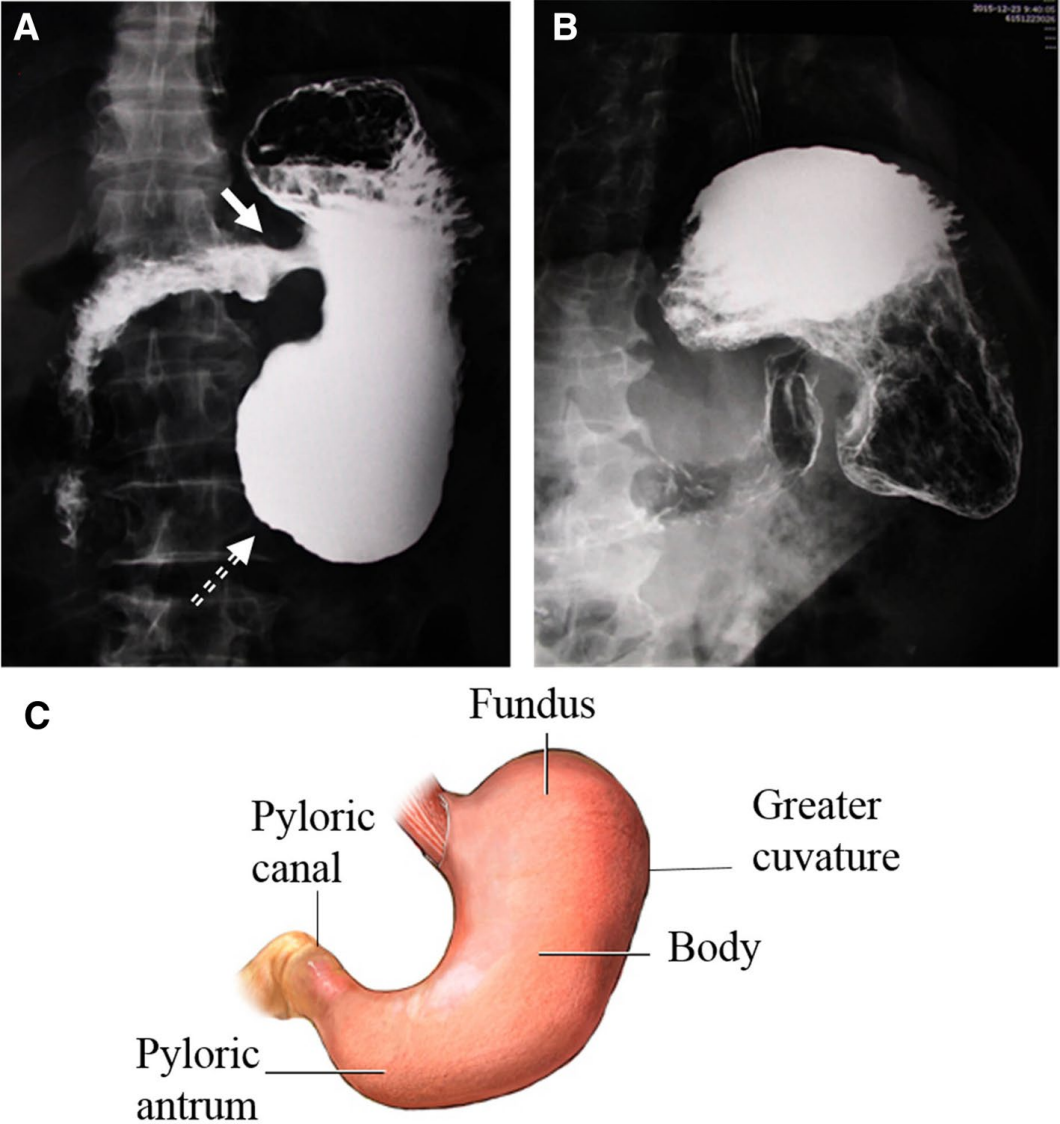
Double-contrast examination of the stomach. Fluid accumulation was shown in the stomach, and the long axis of the stomach was shortened. The pylorus opened to the side of the lesser curvature of the stomach (solid arrow). The distal end of the stomach was enlarged (dashed arrow). The stomach was of “hammer” shape (**a**) as compared to a normal anatomical structure of stomach (**c**). The double-contrast examination showed a bilateral sign on the small curvature of the gastric mucosa, with a fixed shape and partially absent in gastric antrum (**b**)

On the same day of admission, double-contrast examination was performed. Fluid accumulation was observed in the stomach. The long axis of the stomach was shortened. The pylorus was opened to the side of the lesser curvature of the stomach and the contrast agent entered the duodenum from here. The distal end of the stomach was enlarged. The stomach was of “hammer” shape, with the fundus, the body of stomach and the enlarged antrum as the head, and the duodenum as the handle (Fig. 1a). No other congenital abnormalities were detected.

The examination also showed a bilateral sign on the small curvature of the gastric mucosa, with a fixed shape, partially absent in the gastric antrum (Fig. 1b). In summary, abnormal pyloric opening in the stomach body and lesions on the lesser curvature was found.

### Gastrointestinal endoscopy

**Fig. 2:**
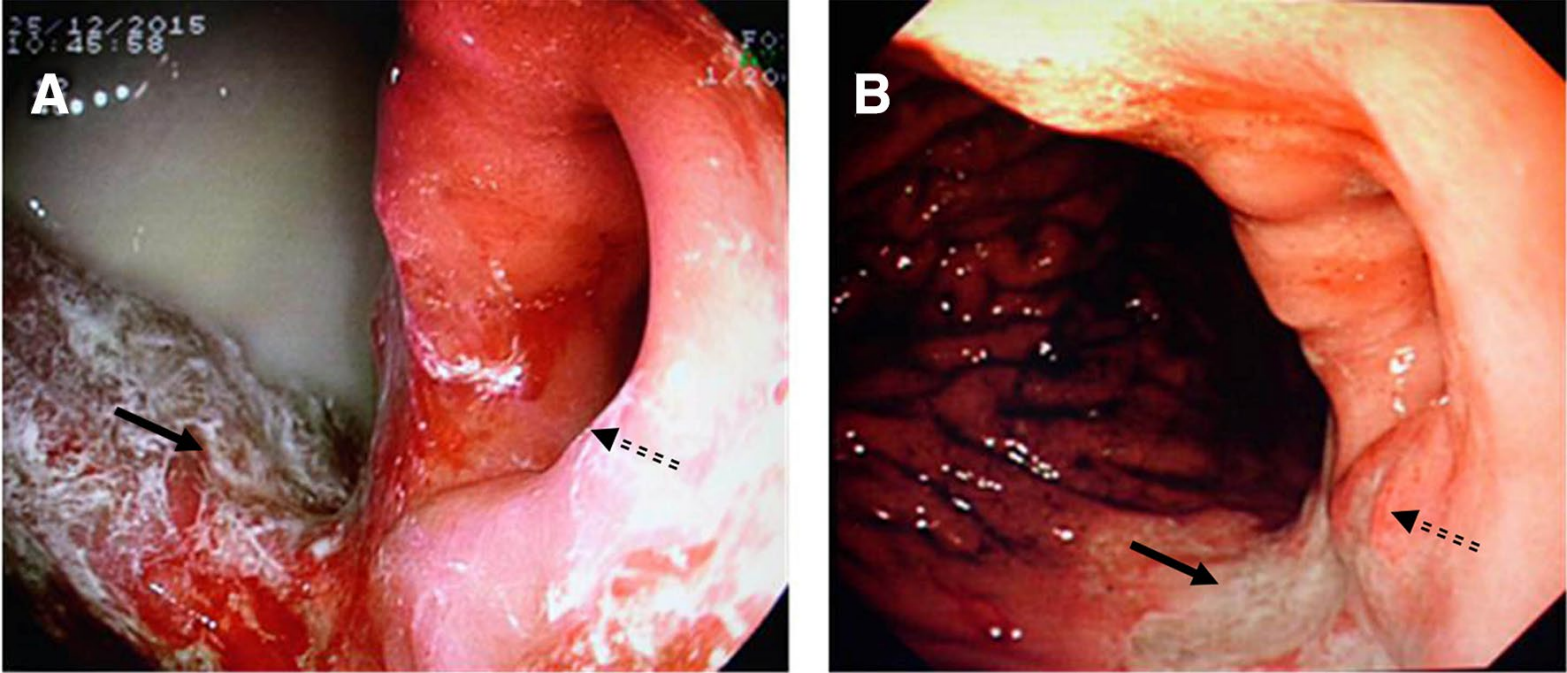
The gastric endoscopy of the stomach. Milky gastric contents were seen in the gastric lumen and patchy residual barium on the gastric wall. The pyloric antrum (solid arrow) was approximately 3 cm below the cardia (dashed arrow) (**a**). A large ulcer with whitish exudate covering the base was found on the posterior wall. The border of the ulcer was not clear and poorly elastic (solid arrow). Around the ulcer, there’s rough and brittle mucosal uplift (dashed arrow). The pyloric open was round with poor function (**b**)

Gastrointestinal endoscopy was performed 2 days after the double-contrast examination. A large amount of milky gastric contents were observed in the gastric lumen and patchy residual barium was found on the gastric wall. The pyloric antrum was approximately 3 cm below the cardia (Fig. 2a).

A second gastrointestinal endoscopy was performed 2 weeks later. Under endoscopy, the gastric fundal mucosa was shown to be congested with edema. A large number of brown stomach contents were observed in the stomach. The stomach body and the gastric antrum were shortened. The gastric antrum was a cystic blind end. A large ulcer with whitish exudate covering the base was found on the posterior wall, and the border of the ulcer was not clear and poorly elastic. Around the ulcer, there is rough and brittle mucosal uplift and biopsy was done. The pyloric antrum was located at the upper lesser curvature. The pyloric open was round with poor function. No obvious abnormalities in the bulb and descending part of the duodenum were observed (Fig. 2b).

Rough histological examination of the specimen showed broken mucosal glands with atypical hyperplasia and focal carcinogenesis. Detailed histological examination and surgery were recommended but refused by the patient.

## Discussion and conclusions

In this case report, a pyloric ectopic opening in the stomach body with rough histologic examination is presented.

Stomach is the most dilated part of the digestive tube. Three types of stomach may persist clinically, which are hypersthenic, sthenic and hyposthenic types [4]. The shape of the stomach could vary frequently without any clinical symptoms. Burdan et al. classified the anatomical variations of the stomach into five primary groups, which are abnormal position along longitudinal or horizontal axis, abnormal shape, abnormal stomach connections, and mixed forms [2]. Another study on the shape and topography of stomach anatomical classification grouped the anatomical variation of stomach as herniated, malrotated and congenital variants [1]. Normally, pylorus opens in the distal antrum with a length of about 5 mm. Abnormality of pylorus including double pylorus and congenital pyloric stenosis has been reported [5, 13]. In this case, the pylorus was approximately just 3 cm below the cardia and had the opening high at the lesser curvature, making the stomach in a “hammer” shape, which has not been reported before.

The variation of stomach shape and pylorus may be congenital or acquired later in life. Nayak et al. suggested that a rare case of “hourglass”-shaped stomach with the presence of an unusual incisure at the greater curvature was probably of congenital origin [11]. Rollins et al. found that pyloric stenosis was usually not present at birth and probably develops afterward [12] (Table 1). Other factors such as feeding habits may reversibly affect the stomach shape [7, 10]. In this case, the patient denied any history of stomach surgery, which ruled out the possibility the variation was caused by surgery. There was a large ulcer in the gastric antrum. The scar contraction of the gastric ulcer could potentially cause abnormal stomach shape. The scar contraction may shorten the gastric curvature and make the pylorus close to the cardia. However, in this case, no scar contraction was observed in endoscopy. The pyloric ectopic opening is probably congenital.

**Table 1:** Variations of stomach anatomy

Variations	Anomalies
Hypertrophic pyloric stenosis [4]	Elongation of the pyloric channel, indentation of both duodenal bulb and gastric antrum by the pyloric mass, and gastric hyperperistalsis that stops abruptly at the pylorus.
Double pylorus [6]	Fistulous communication between the gastric antrum and the duodenal bulb.
“Hourglass” stomach [7]	The stomach had two distinct pouches. The two pouches were formed due to the presence of an unusually deep notch at the greater curvature. Both the pouches communicated with the distal end of the esophagus.

Variation in stomach shape and pyloric opening may be with some clinical disturbance. The ectopic pylorus raised the outflow tract and may result in delayed gastric emptying. Chronically, gastroptosis and decreased gastric motility may occur and patients may complain about abdominal distention or vomit after meal. Studies have shown that *Helicobacter pylori* infection might delay the gastric emptying [3, 9] and the decreased gastric motility in these patients might result in complications. In Einhorn et al.’s work, double pylorus is associated with symptoms suggesting peptic ulcers, and a small part of patients reported gastric bleeding [5]. They also showed 80% of patients responded well to medical therapy and were free of complications [5]. For 20% of anatomical variations that cause complication, correcting for the variation may benefit the symptoms [8]. In this case, non-ectopic pylorus-related symptoms were presented. However, it is possible that the delayed gastric emptying may worsen the HP infection and ulcer. Unfortunately, the HP test was refused by the patient.

In summary, this case showed a pyloric ectopic opening in the gastric body with a “hammer” shape stomach. This type of stomach may be unnoticed throughout patients’ life without any complication. Our case adds a new gastric morphological variation, which may be important to radiologist in the interpretation of double-contrast examination.
